# Association of changes in body weight and waist circumference with a subsequent risk of developing hypertension in men requiring specific healthcare guidance

**DOI:** 10.1038/s41440-025-02397-4

**Published:** 2025-10-24

**Authors:** Yuya Kitani, Yuta Suzuki, Hidehiro Kaneko, Akira Okada, Hiroyuki Morita, Katsuhito Fujiu, Koichi Node, Hideo Yasunaga, Norihiko Takeda, Naoki Nakagawa

**Affiliations:** 1 Division of Cardiology and Nephrology, Department of Internal Medicine, Asahikawa Medical University, Hokkaido, Japan; 2 Department of Cardiovascular Medicine, The University of Tokyo, Tokyo, Japan; 3 Center for Outcomes Research and Economic Evaluation for Health, National Institute of Public Health, Saitama, Japan; 4 The Department of Advanced Cardiology, The University of Tokyo, Tokyo, Japan; 5 Department of Prevention of Diabetes and Lifestyle-Related Diseases, Graduate School of Medicine, The University of Tokyo, Tokyo, Japan; 6 Department of Cardiovascular Medicine, Saga University, Saga, Japan; 7 Department of Clinical Epidemiology and Health Economics, School of Public Health, The University of Tokyo, Tokyo, Japan

**Keywords:** body weight change, waist circumference change, hypertension, prevention

## Abstract

Although body weight (BW) and waist circumference (WC) reduction are key goals of Japan’s Specific Health Guidance program, limited evidence exists linking these reductions to a lower incidence of hypertension. This study aimed to evaluate the association between changes in BW and WC and the subsequent development of hypertension among men in a nationwide population. We retrospectively analyzed 23,109 men aged 40–64 years who required intensive health guidance and had no prior history of hypertension, using a nationwide database (DeSC Healthcare, Tokyo, Japan) from April 2014 to August 2023. One-year changes in BW and WC were examined for their association with incident hypertension using multivariable Cox regression and cubic spline analyses. During a mean follow-up of 1381 ± 789 days, 4162 men (18.0%) developed hypertension. Greater reductions in BW and WC were associated with a progressively lower risk of hypertension. In multivariable Cox models, BW reductions of ≤ −3.0 kg, −2.9 to −2.0 kg, and −1.9 to −1.0 kg were significantly associated with reduced hypertension risk (HR: 0.74 [95% CI: 0.67–0.82], 0.85 [0.76–0.96], and 0.89 [0.81–0.99], respectively). WC reductions of ≤ -3.0 cm and −2.9 to −2.0 cm were also significantly associated with reduced risk (HR: 0.80 [0.73–0.88] and 0.81 [0.72–0.92], respectively). Cubic spline analyses confirmed a monotonic decrease in hypertension risk with increasing BW and WC reduction. Among men eligible for Specific Health Guidance, one-year reductions in BW and WC were significantly associated with a lower risk of developing hypertension.

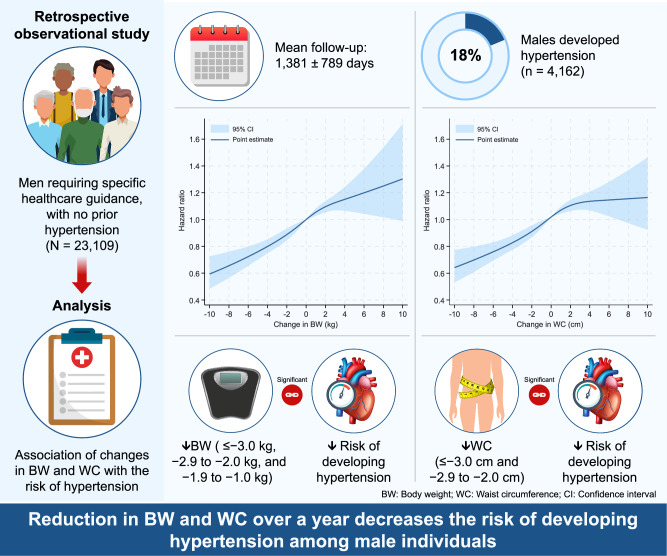

## Introduction

In the fourth phase of the Specific Health Checkups and Specific Health Guidance program [1, 2], which commenced in 2024, a greater emphasis was placed on reducing body weight (BW) and waist circumference (WC). The overarching goal of this program is to prevent lifestyle-related diseases, particularly hypertension and cardiovascular diseases (CVDs). Hypertension, as the most prevalent lifestyle-related disease, remains one of the strongest risk factors for CVDs [3, 4]. While BW and WC reduction are recognized as critical objectives within the program [1], there is limited empirical evidence linking these reductions to a decreased incidence of hypertension [5, 6]. This study aims to address this gap by leveraging a large-scale epidemiological dataset to investigate whether reductions in BW and WC in men targeted for Specific Health Guidance are associated with a lower incidence of hypertension.

Point of view

- Clinical relevance
  One-year reduction in body weight and waist circumference is associated with a lower risk of developing hypertension in high-risk men.
- Future direction
  Future studies should focus on long-term adherence and behavior change and extend analyses to women to enhance generalizability.
- Consideration for the Asian population
  The study provides evidence supporting Japan’s national health guidance program, highlighting the value of modest, sustained reductions in body weight and waist circumference.

## Methods

### Study design

This study had a retrospective observational design and analyzed data from the DeSC database, which contains large-scale claims and medical check-up data between April 2014 and August 2023 provided by DeSC Healthcare (Tokyo, Japan) [7]. The DeSC database is a collection of annual health check-up records including data on blood pressure (BP) measurements, fasting blood examinations, medical history, current medications, and the individuals’ responses to a standard questionnaire of lifestyle habits. The DeSC database covers three major health insurance systems in Japan: the health insurance for employees of large companies (Kempo), the National Health Insurance for nonemployees (Kokuho), and the Advanced Elderly Medical Service System for older people aged ≥ 75 years (Koki Koreisha Iryo Seido). This broad coverage allows the DeSC database to be highly representative of the Japanese population across different age groups and socio-economic statuses. The comprehensive nature of the DeSC database strengthens the validity of our findings by ensuring a complete and more representative sample of the Japanese population. Annual health check-ups are mandatory for the general population in Japan, where more than 70% of men aged 45–54 years undergo health check-ups at least once a year [8].

There are two types of Specific Health Guidance in the fourth phase of the Specific Health Checkups and Specific Health Guidance program [1]. The intensive health guidance program is offered to those who have two or more risk factors with abdominal obesity or three or more risk factors with overweight (BMI ≥ 25 kg/m^2^) but without abdominal obesity. Abdominal obesity is defined as waist circumference ≥ 85 cm in men. The four risk factors are (1) hemoglobin A1c [HbA1c]≥5.6%, (2) triglycerides≥150 mg/dl and/or high-density lipoprotein cholesterol<40 mg/dl, (3) systolic blood pressure≥130 mmHg and/or diastolic blood pressure≥85 mmHg, (4) smoking status. Another program, referred to as the motivational health guidance program, is offered to individuals who have one risk factor with abdominal obesity or two risk factors with overweight without abdominal obesity. In this study, we focused on individuals classified as in need of specific healthcare guidance (active support in the intensive health guidance program) without a prior diagnosis of hypertension and without the use of blood pressure-lowering medications.

### Ethics statement

The study was approved by the Ethics Committee of the University of Tokyo (approval number:2021010NI) and conducted in accordance with the Declaration of Helsinki. The requirement for informed consent was waived because all data included in the dataset were de-identified.

### Study participants

**Fig. 1:**
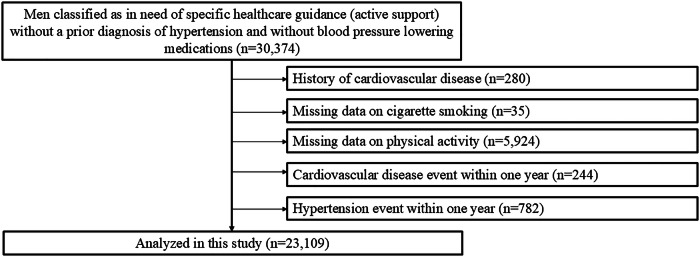
Flowchart. We identified 30,374 men classified as in need of specific healthcare guidance (active support) without a prior diagnosis of hypertension and without BP lowering medications in the DeSC database. We excluded individuals for the following reasons: a prior history of cardiovascular disease; missing data on cigarette smoking and physical activity; cardiovascular disease event within one year; and hypertension event within one year. Finally, we analyzed 23,109 participants

We analyzed data for 30,374 men aged 40-64 years who were enrolled in the DeSC database between April 2014 and August 2023 and classified as in need of specific healthcare guidance (active support in the intensive health guidance program) without a prior diagnosis of hypertension and without blood pressure-lowering medications. Individuals with a history of cardiovascular disease (*n* = 280), with missing data on cigarette smoking (*n* = 35) and physical activity (*n* = 5924), with cardiovascular disease event within one year (*n* = 244), and with hypertension event within one year (*n* = 782) were excluded. Finally, we assessed the association of one-year change of BW and WC with the incidence of hypertension in 23,109 men (Fig. 1).

### Measurements and definitions

We obtained the data of body mass index (BMI), WC, BP and laboratory data (HbA1c, low-density lipoprotein cholesterol, high-density lipoprotein cholesterol, and triglycerides). Information on history of smoking (current or non-current) and physical activity (active or inactive) was obtained from a self-reported questionnaire completed during the health check-up. Physical activity was defined as 30 min of exercise at least twice a week or ≥ 1 h of walking per day [9]. In the Japanese health check-up system, healthcare professionals measure the BP using a standard sphygmomanometer or an automated device at least twice after the participant has been in a resting condition, and the average is recorded [10, 11].

We defined diabetes as an HbA1c ≥ 6.5% or use of blood glucose-lowering medications and dyslipidemia as low-density lipoprotein cholesterol≥140 mg/dL, high-density lipoprotein cholesterol<40 mg/dL, triglycerides ≥ 150 mg/dL, or use of lipid-lowering medications.

### Outcome

The primary outcome was incident hypertension (ICD-10 codes I10-I15) [12]. Study participants were followed until the incidence of the outcome, loss of insurance coverage, death, or study end date (August 2023).

### Statistical analysis

Continuous variables were presented as the median (25–75th percentiles) and categorical variables as the number (percentage). We conducted multivariable Cox regression analyses to assess the association of changes in BW and WC with a subsequent risk of developing hypertension. Hazard ratios (HRs) and 95% confidence intervals (CIs) for developing hypertension were calculated for each value of change in BW using a BW change category (≤ −3.0 kg, −2.9 to −2.0 kg, −1.9 to −1.0 kg, −0.9 to 0.9 kg, 1.0 to 1.9 kg, 2.0 to 2.9 kg, 3.0 kg ≤) and for each value of change in WC using a WC change category (≤ −3.0 cm, −2.9 to −2.0 cm, −1.9 to −1.0 cm, −0.9 to 0.9 cm, 1.0 to 1.9 cm, 2.0 to 2.9 cm, 3.0 cm ≤) after adjustment for age, BMI, WC, systolic BP, diastolic BP, diabetes, dyslipidemia, smoking, and physical inactivity.

To validate the robustness of our primary findings, we examined the association of changes in BW and WC with a subsequent risk of developing hypertension using a restricted cubic spline regression model with four knots (5, 35, 65, and 95 percentiles). Hazard ratios were adjusted for age, BMI, WC, systolic BP, diastolic BP, diabetes, dyslipidemia, smoking, and physical inactivity.

In a sensitivity analysis, the outcome was redefined as a diagnosis of hypertension with a prescription for antihypertensive medications in the months before and after the diagnosis.

All analyses were performed using STATA version 19 (StataCorp LLC, College Station, TX, USA). A *p*-value < 0.05 was considered statistically significant.

## Results

### Basic characteristics

Table 1 summarizes the basic characteristics of the study participants. The median age for the entire study cohort was 50 years (25–75th percentiles: 45–57 years). The median BW and WC at the initial health checkup was 76.1 (25–75th percentiles: 71.0–82.4), and 90.9 (25–75th percentiles: 87.5–95.3), respectively. The median systolic and diastolic BP were 125 (25–75th percentiles: 117–132) mmHg and 79 (25–75th percentiles: 72–84) mmHg, respectively.

**Table 1:** Baseline characteristics

	Men (n = 23,109)
Age, years	50 (45–57)
Body weight, kg	76.1 (71.0–82.4)
Body mass index, kg/m^2^	25.9 (24.5–27.8)
Obesity, *n* (%)	15,590 (67.5)
Waist Circumference, cm	90.9 (87.5-95.3)
Waist Circumference ≥ 85 cm, *n* (%)	22,801 (98.7)
Systolic blood pressure, mmHg	125 (117–132)
Diastolic blood pressure, mmHg	79 (72–84)
Diabetes mellitus, *n* (%)	1797 (7.8)
Dyslipidemia, *n* (%)	19,214 (83.1)
Cigarette smoking, *n* (%)	14,181 (61.4)
Physical inactivity, *n* (%)	12,552 (54.3)
Hemoglobin A1c, %	5.7 (5.5–5.9)
Low-density lipoprotein cholesterol, mg/dL	135 (115–156)
High-density lipoprotein cholesterol, mg/dL	47 (40–55)
Triglycerides, mg/dL	173 (123–234)

Continuous variables were presented as the median (25–75th percentiles) and categorical variables as the number (percentage)

### Risk of developing hypertension associated with changes in BW and WC

The mean follow-up duration was 1381 ± 789 days, During the observational period, 4162 individuals developed hypertension. The multivariable Cox regression analysis showed that BW reductions of ≤ -3.0 kg, −2.9 to −2.0 kg, and −1.9 to −1.0 kg were significantly associated with the decreased risk of developing hypertension (HR: 0.74, 95% CI: 0.67-0.82, HR: 0.85, 95% CI: 0.76–0.96, and HR: 0.89, 95% CI: 0.81–0.99, respectively) (Table 2A) and the BW gain of≥3.0 kg was significantly associated with the increased risk of developing hypertension (HR: 1.17, 95% CI:1.05–1.31). WC reductions of ≤ −3.0 cm and −2.9 to −2.0 cm were significantly associated with the decreased risk of developing hypertension (HR: 0.80, 95% CI: 0.73-0.88, and HR: 0.81, 95% CI: 0.72–0.92, respectively) (Table 2B) and WC gains of 2.0 to 2.9 cm and ≥ 3.0 cm tend to be associated with the increased risk of developing hypertension (HR: 1.11, 95% CI:0.99–1.24, and HR: 1.09, 95% CI: 0.99–1.20, respectively). The risk of developing hypertension decreased monotonically as BW and WC were reduced, and became significantly lower when BW and WC decreased below −1 kg and −2 cm, respectively (Table 2). When annual changes in either body weight (BW, per 1 kg increase per year) or waist circumference (WC, per 1 cm increase per year) were individually included in regression analyses, the HR (95% CI) associated with a 1 kg/year increase in BW was 1.05 (1.04–1.06; *P* < 0.001), and the HR (95% CI) associated with a 1 cm/year increase in WC was 1.04 (95% CI: 1.03–1.04; *P* < 0.001). When each variable was included in the regression analysis at the same time, the HR (95% CI) associated with a 1 kg/year increase in BW was 1.03 (1.02–1.05; *P* < 0.001) and the HR (95% CI) associated with a 1 cm/year increase in WC was 1.02 (1.00–1.03; *P* = 0.021).

**Table 2:** Risk of Developing Hypertension

	Number	Number of Events	Incidence Per 10,000 person-years (95% CI)	Hazard Ratio (95% CI)
Change in Body weight (kg)				
≤ −3.0	3244	445	377.1 (343.6–413.8)	0.74 (0.67–0.82)
−2.9 to −2.0	1966	327	439.1 (394.0–489.4)	0.85 (0.76–0.96)
−1.9 to −1.0	3035	535	462.8 (425.2–503.7)	0.89 (0.81–0.99)
−0.9 to 0.9	7496	1429	496.6 (471.5–523.0)	1 (Ref)
1.0 to 1.9	3225	616	495.2 (457.6–535.9)	1.03 (0.93–1.13)
2.0 to 2.9	1903	368	510.0 (460.5–564.9)	1.06 (0.95–1.19)
3.0 ≤	2240	442	543.0 (494.7–596.1)	1.17 (1.05–1.31)
(B) Change in Waist Circumference (cm)				
≤ −3.0	4750	714	408.7 (379.8–439.8)	0.80 (0.73–0.88)
−2.9 to −2.0	2110	330	406.9 (365.3–453.3)	0.81 (0.72–0.92)
−1.9 to −1.0	2685	500	477.1 (437.1–520.8)	0.95 (0.85–1.05)
−0.9 to 0.9	5426	1014	488.8 (459.6–519.8)	1 (Ref)
1.0 to 1.9	2643	502	499.0 (457.2–544.6)	1.02 (0.92–1.14)
2.0 to 2.9	1967	406	544.9 (494.4–600.5)	1.11 (0.99–1.24)
3.0 ≤	3528	696	532.7 (494.6–573.8)	1.09 (0.99–1.20)

We performed the multivariable Cox regression analyses including age, body mass index, waist circumference, systolic blood pressure, diastolic blood pressure, diabetes, dyslipidemia, smoking, and physical inactivity. Hazard ratios (95% confidence interval [95% CI]) are presented

**Fig. 2:**
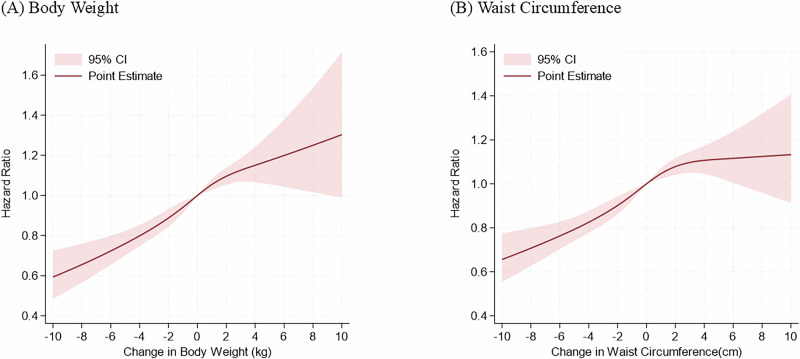
Restricted cubic spline showing hazard ratios for the development of hypertension along with continuous body weight (BW) change (**A**) and waist circumference (WC) change (**B**) in men. Restricted cubic spline with four knots (5, 35, 65 and 95 percentiles) shows the relationship of change in BW and WC with development of hypertension. Hazard ratios are adjusted for age, body mass index, waist circumference, systolic BP, diastolic BP, diabetes, dyslipidemia, smoking, and physical inactivity

In the restricted cubic spline function, the risk of developing hypertension continued to decrease monotonically as the reduction in BW and WC increased (Fig. 2). Furthermore, the Cox regression model including the interaction term between continuous changes in BW and WC for the risk of developing hypertension is shown in Figure S1 (interaction P-value = 0.3248). Although there is no significant interaction, it suggests that increases in both BW and WC raise the risk of developing hypertension, and conversely, decreases in both may reduce the risk of developing hypertension.

In the sensitivity analysis in which the outcome was restricted to a combination of antihypertensive prescription and hypertension diagnosis, BW reduction of ≤ −3.0 kg was associated with the decreased risk of developing hypertension (HR: 0.72, 95% CI: 0.63–0.82) (Table 3A). Men with WC reductions of ≤ −3.0 cm and −2.9 to −2.0 cm had a lower risk of developing hypertension (HR: 0.76, 95% CI: 0.67–0.86, and HR: 0.78, 95% CI: 0.67–0.91, respectively) (Table 3B).

**Table 3:** Risk of Developing Hypertension after Redefining the Outcome

	Number	Number of Events	Incidence Per 10,000 person-years (95% CI)	Hazard Ratio (95% CI)
Change in Body weight (kg)				
≤ −3.0	3082	283	248.6 (221.2–279.3)	0.72 (0.63–0.82)
−2.9 to −2.0	1859	220	306.6 (268.6–349.9)	0.87 (0.75–1.01)
−1.9 to −1.0	2866	366	328.8 (296.8–364.2)	0.92 (0.82–1.04)
−0.9 to 0.9	7001	934	339.0 (318.0–361.5)	1 (Ref)
1.0 to 1.9	3005	396	332.9 (301.7–367.3)	1.02 (0.91–1.15)
2.0 to 2.9	1784	249	359.4 (317.4–406.9)	1.11 (0.96–1.27)
3.0 ≤	2100	302	386.4 (345.1–432.5)	1.24 (1.09–1.42)
(B) Change in Waist Circumference (cm)				
≤ −3.0	4488	452	269.2 (245.5–295.2)	0.76 (0.67–0.86)
−2.9 to −2.0	1994	214	273.6 (239.3–312.8)	0.78 (0.67–0.91)
−1.9 to −1.0	2517	332	330.2 (296.6–367.8)	0.94 (0.82–1.07)
−0.9 to 0.9	5088	676	339.7 (315.0–366.3)	1 (Ref)
1.0 to 1.9	2487	346	356.6 (320.9–396.2)	1.05 (0.93–1.20)
2.0 to 2.9	1831	270	380.9 (338.1–429.1)	1.11 (0.96–1.28)
3.0 ≤	3292	460	367.4 (335.3–402.5)	1.08 (0.96–1.22)

We performed the multivariable Cox regression analyses including age, body mass index, waist circumference, systolic blood pressure, diastolic blood pressure, diabetes, dyslipidemia, smoking, and physical inactivity. Hazard ratios (95% confidence interval [95% CI]) are presented

## Discussion

This study included 23,109 male participants requiring specific healthcare guidance (active support in the intensive health guidance program) with normal BP at the initial health checkup, demonstrating the association of changes in BW and WC with a subsequent risk of developing hypertension. The risk of developing hypertension continued to decrease monotonically as the reduction in BW and WC increased. Multivariable Cox regression analysis showed the risk of developing hypertension significantly decreased when the BW and WC fell below −1 kg and −2 cm, respectively.

In Japan, the Specific Health Checkup and Specific Health Guidance were implemented in 2008 to reduce medical costs in the medium to long term by preventing metabolic syndrome and subsequent cardiovascular disease [1]. Our study showed that BW and WC reduction in high-risk individuals for metabolic syndrome were associated with a decrease in subsequent risk of developing hypertension. This aligns with existing evidence that links BW decrease to BP reduction [1, 9, 13, 14]. However, the association between WC reduction and hypertension risk was less clear. Several studies suggest that while WC is a strong marker of visceral fat and metabolic risk [14, 15], its impact on hypertension may be modulated by other factors such as insulin resistance and endothelial dysfunction [16, 17].

Importantly, our findings underscore that even modest BW loss—approximately 1 kg over one year—was significantly associated with decreased hypertension risk. This contrasts with the current Specific Health Guidance goal of achieving a 2 kg BW loss over three months [1, 13]. The evidence suggests that gradual, sustained BW reduction may be rather effective in managing hypertension risks, highlighting the importance of long-term BW management strategies. Furthermore, the HR for incident hypertension showed a steeper downward slope with decreases in body weight and waist circumference than the upward slope observed with increases, indicating a stronger preventive effect of reductions than the harmful effect of comparable increases.

Our study has the following strengths and novelties by analyzing the large, population-based cohort of nonhypertensive adults requiring specific healthcare guidance to examine the association between one-year changes in BW and WC and hypertension development. So far, there have been no concrete data on the risk of developing hypertension among high-risk individuals for metabolic syndrome without hypertension. Our study is distinguishable from preceding studies in that the association of one-year changes in BW and WC with a subsequent risk of developing hypertension in men was clearly demonstrated, highlighting a potential for optimizing modifiable risk factors and lifestyles to prevent hypertension development in nonhypertensive men requiring specific healthcare guidance. Furthermore, in the fourth phase of the Specific Health Checkups and Specific Health Guidance program [1, 2], which commenced in 2024, a greater emphasis was placed on reducing BW and WC. Individuals who have shown improvement through the Specific Health Guidance program correspond to those with a BMI < 30 kg/m^2^ who experienced a decrease of at least 1.0 cm in waist circumference and 1.0 kg in body weight in the second year compared to the first year [2]. Therefore, our findings could significantly support the validity of the new specific healthcare guidance, particularly the active support provided in the intensive health guidance program.

Despite these insights, this study has several limitations. First, it could not differentiate the mechanisms underlying BW loss, such as dietary changes or increased physical activity. Second, while participants were identified as eligible for Specific Health Guidance, we lacked data on whether the guidance was actually implemented or adhered to, potentially making a bias inevitable. Furthermore, the reliance on single-point measurements for BW and WC during annual checkups raises concerns about measurement precision and variability [1, 13]. Future research should focus on prospective, longitudinal data collection to clarify the causal pathways between BW/WC reduction and preventing hypertension. Additionally, refining Specific Health Guidance programs to prioritize long-term adherence and behavior change could further enhance their efficacy in preventing hypertension and related complications [1, 6]. Our study is unique in that it examined BW and WC reduction over one year, whereas the Specific Health Guidance programs aims to achieve a 2 kg-BW loss over three months. While the present study focused on changes in body weight or waist circumference in relation to the risk of developing hypertension, future studies may justify the consideration of Specific Health Guidance informed by percentage changes in BMI and WC. Finally, we excluded female participants because of the potential for hormonal fluctuations as well as age-related change in hormonal environment (e.g., menopause) to affect vascular function. This decision was consistent with previous research suggesting that estrogen, particularly during the follicular phase of the menstrual cycle, enhances vasodilation and endothelial function by increasing nitric oxide availability [17]. However, the exclusion of women reduces the applicability of our findings to the general population. Inclusion of both sexes in future research would increase the generalizability of these findings.

### Perspective of Asia

The relationship between body composition and anthropometric indices differs between Asian and Caucasian populations. Therefore, an obesity index that is useful for Caucasians may not necessarily be useful for Asians. The role of obesity in the development of hypertension may also differ between ethnic groups. Further longitudinal studies are needed to examine the effects of visceral obesity and body composition on the development of hypertension and cardiovascular events in Asian populations.

In conclusion, our analysis using a large-scale epidemiological dataset revealed that a one-year reduction in BW and WC was significantly associated with the decreased risk of developing hypertension among male individuals eligible for Specific Health Guidance.

## Supplementary information

Supplementary Information

## Data Availability

This database is available for anyone who purchases it from DeSC Healthcare (Tokyo, Japan).
