# Correction: Association of changes in body weight and waist circumference with a subsequent risk of developing hypertension in men requiring specific healthcare guidance

**DOI:** 10.1038/s41440-025-02457-9

**Published:** 2025-11-14

**Authors:** Yuya Kitani, Yuta Suzuki, Hidehiro Kaneko, Akira Okada, Hiroyuki Morita, Katsuhito Fujiu, Koichi Node, Hideo Yasunaga, Norihiko Takeda, Naoki Nakagawa

**Affiliations:** 1 Division of Cardiology and Nephrology, Department of Internal Medicine, Asahikawa Medical University, Hokkaido, Japan; 2 Department of Cardiovascular Medicine, The University of Tokyo, Tokyo, Japan; 3 Center for Outcomes Research and Economic Evaluation for Health, National Institute of Public Health, Saitama, Japan; 4 The Department of Advanced Cardiology, University of Tokyo, Tokyo, Japan; 5 Department of Prevention of Diabetes and Lifestyle-Related Diseases, Graduate School of Medicine, University of Tokyo, Tokyo, Japan; 6 Department of Cardiovascular Medicine, Saga University, Saga, Japan; 7 Department of Clinical Epidemiology and Health Economics, School of Public Health, University of Tokyo, Tokyo, Japan

Correction to: *Hypertension Research*

10.1038/s41440-025-02397-4, published online 24 October 2025

In this article, Hideo Yasunaga was incorrectly denoted as the corresponding author, but it should have been Hidehiro Kaneko. The original article has been corrected.

