# Supplementary material for: Association of changes in body weight and waist circumference with a subsequent risk of developing hypertension in men requiring specific healthcare guidance
Source: Hypertens Res. 2025 Oct 24;49(2):508–15. doi: 10.1038/s41440-025-02397-4 (PMC12823394; doi:10.1038/s41440-025-02397-4)
Supplement: Supplementary file 1 — Supplementary Information [file 41440_2025_2397_MOESM1_ESM.docx]

**Association of changes in body weight and waist circumference with a subsequent risk of developing hypertension in men**

Yuya Kitani ^1^, Yuta Suzuki ^2,3^, Hidehiro Kaneko ^2^, Akira Okada ^5^, Hiroyuki Morita ^2^, Katsuhito Fujiu ^2,6^, Koichi Node ^7^, Hideo Yasunaga ^8^, Norihiko Takeda ^2^, Naoki Nakagawa ^1^

^1^Division of Cardiology and Nephrology, Department of Internal Medicine, Asahikawa Medical University, Hokkaido, Japan

^2^Department of Cardiovascular Medicine, The University of Tokyo, Tokyo, Japan

^3^Center for Outcomes Research and Economic Evaluation for Health, National Institute of Public Health, Saitama, Japan

^4^The Department of Advanced Cardiology, The University of Tokyo, Tokyo, Japan

^5^Department of Prevention of Diabetes and Lifestyle-Related Diseases, Graduate School of Medicine, The University of Tokyo, Tokyo 113-0013, Japan.

^6^Department of Advanced Cardiology, The University of Tokyo, Tokyo 113-0013, Japan.

^7^Department of Cardiovascular Medicine, Saga University, Saga 840-8502, Japan.

^8^Department of Clinical Epidemiology and Health Economics, School of Public Health, The University of Tokyo, Tokyo 113-0013, Japan.

Correspondence: Hidehiro Kaneko, Department of Cardiovascular Medicine, Graduate School of Medicine, The University of Tokyo, 7- 3- 1 Hongo, Bunkyo- ku, Tokyo113- 8655, Japan.

e-mail: kanekohidehiro@gmail.com

**Supplemental Figure 1**

Figure S1. The risk of developing hypertension predicted by continuous body weight change and waist circumference change using contour plots after adjusted for age, body mass index, waist circumference, systolic BP, diastolic BP, diabetes, dyslipidemia, smoking, and physical inactivity. The each curve indicates the same odds ratio.
